# Contributions from Mexican Flora for the Treatment of Diabetes Mellitus: Molecules of *Psacalium decompositum* (A. Gray) H. Rob & Brettell

**DOI:** 10.3390/molecules26102892

**Published:** 2021-05-13

**Authors:** Manuel Jiménez-Estrada, Maira Huerta-Reyes, Rosario Tavera-Hernández, J. Javier Alvarado-Sansininea, Ana Berenice Alvarez

**Affiliations:** 1 Instituto de Química, Universidad Nacional Autónoma de México, Ciudad Universitaria, Ciudad de México, Coyoacán 04510, Mexico; rosario.tavera@gmail.com (R.T.-H.); javier33@comunidad.unam.mx (J.J.A.-S.); aniiitarez@gmail.com (A.B.A.); 2 Unidad de Investigación Médica en Enfermedades Nefrológicas, Hospital de Especialidades “Dr. Bernardo Sepúlveda Gutiérrez”, Centro Médico Nacional Siglo XXI, Instituto Mexicano del Seguro Social, Cuauhtémoc 06720, Mexico; chilanguisima@yahoo.com

**Keywords:** Mexican medicinal plants, hypoglycemic, *Psacalium decompositum*, sesquiterpenes, cacalol, cacalone, diabetes mellitus, antioxidant, anti-inflammatory

## Abstract

Diabetes mellitus (DM) is cited as a serious worldwide health problem that occupies second place in causes of annual mortality in Mexico. Among Mexican flora, nearly 300 plant species have been employed as hypoglycemic in popular use. Thus, their study entertains great relevance In this context, this work contributes a clear and timely review of the plant species utilized in Traditional Mexican Medicine and experimental biological models in which not only have the hypoglycemic properties of the extracts and the isolated compounds been considered, but also the anti-inflammatory and antioxidant properties, taking into account an integral focus based on the complex mechanisms involved in the pathogenesis and physiopathology of DM. Among the species reviewed, we highlight *Psacalium decompositum* (Asteraceae), due to the potent hypoglycemic, anti-inflammatory, and antioxidant activity of the sesquiterpenes identified as majority compounds isolated from the root, such as cacalol and cacalone that also possess the capacity of increasing insulin levels. In this manner, the present manuscript attempts to contribute necessary information for the future study of bioactive molecules that are useful in the treatment of DM, as well as also being a contribution to the knowledge and diffusion of Mexican Traditional Medicine.

## 1. Introduction

Diabetes mellitus (DM) has been described as a metabolic disorder characterized by chronic hyperglycemia caused by impaired insulin secretion or impaired insulin action or both [1,2]. DM is a chronic disease with worldwide relevance in view of its impact on the general population, in which it is calculated to be the direct cause of the death of approximately 1.6 million persons worldwide in the year 2016, being the second principal cause of mortality. The International Diabetes Federation (IDF) refers that, at present, 463 million persons around the world have diabetes and that this number is increasing. Therefore, it is projected that, by the year, 2030, this number will have increased to approximately 578 million [1].

DM presents in all age groups regardless of the geographic factor, and it is calculated that more than 1.1 million children and adolescents under that age of 20 years have type 1 DM worldwide, while 3 of every 4 persons with diabetes (352 millions) are found at working age (20–64 years). The increase in prevalence implies a demand for guaranteeing access to specialized medical care, which represents enormous expenses in health, in which, just for the year 2019, it was estimated that DM signified an expenditure of approximately USD 7 billion 60 million dollars. In addition to the latter, DM-associated complications such as diminished vision or blindness, renal insufficiency, and amputation of the lower limbs can generate disability, as well as the fact that suffering from DM increases the risk of cardiopathies, accidents, and myocardial infarcts, causes due to which 50% of patients with DM have died. Thus, this information points to that complications related with DM represent strong damage to patients and their relatives in all ambits of life and, of course, where these represent a considerable economic burden [3].

In the case of Mexico, DM is cited as a grave problem, due to that it continues to occupy second place in causes of mortality at the national level, registering 104,354 deaths, which represent 15.7% of total annual deaths according to the report published in October of that year (2020) by the Mexican National Statistics and Geography Institute [4]. Likewise, the cost of care for diabetes is estimated as above USD 7.7 billion annually, of which approximately USD 4 billion were supplied by the Mexican Ministry of Health (SSA), which cares for the uninsured population; USD 1.2 billion corresponded to the Mexican Institute of Social Security (IMSS) and to the Mexican Institute of Social Security for Services to State Workers (ISSSTE), which provide care for the insured population, while USD 1.8 billion were financed out-of-pocket by the patients themselves and USD 100 million by private health services. These numbers take into account direct costs (medical diagnostic consultations, medicines, hospitalization, retinopathies, cardiovascular diseases, nephropathies, and neuropathies) as indirect expenses (mortality, and temporary or permanent disability) calculated annually [5]. Therefore, due to the transcendence and magnitude of the illness, in the year 2016 the SSA declared DM as an epidemiological emergence in the country [6]. For these reasons, future drugs that can contribute to the control or treatment of DM represent a possible way of improving the quality of life of patients with DM and of their families, and of supplying alternatives in the fight against this disease of worldwide relevance, and especially in Mexican population, which, within a framework of an integral strategy, also covers educative, psychological, and nutritional aspects with the purpose of preventing the development of DM and its complications.

Therefore, among the alternatives for the development of novel medicines that are useful in the treatment of DM, plants are considered an important option, not only due to their active ingredients that can be isolated, but also because of the acceptance of persons concerning the use of plants to treat diseases that, according to data reported by the World Health Organization (WHO), reveal that between 70% and 95% of persons utilize traditional medicines for primary care throughout the world [7], thus situating the investigation of plants for the treatment of diabetes as an attractive, immediate, and necessary activity-to-develop.

### 1.1. Plants in the Treatment of DM

From ancient times, the use has been reported of plants in the treatment of diabetes. A large number of plants have been described for the treatment of DM the world over, and due to the great number of existing ethnobotanical studies, it is difficult to determine the amount of medicinal species employed empirically in the control of this disease [8].

In Mexico, about 300 plant species from 235 genera and 93 families with hypoglycemic effects have been reported [9]; however, Escandón-Rivera and coworkers estimate that at least 800 plants are used for treating DM [10]. The most commonly mentioned families, according to Andrade-Cetto, are Asteraceae (47), Fabaceae (27), Cactaceae (16), Laminaceae (9), and Solanaceae y Euphorbiaceae (10). In the majority of cases, evidence of this action is based purely on experiences with animals or on their traditional use. The latter, however, provides only limited information on their clinical potential, in that the use of hypoglycemic plant remedies is not supported by rigorous clinical assays and requires additional investigation [11]. Various medicinal plant extracts are frequently employed for DM in the majority of cultures. Theoretically, medicinal plants can act as hypoglycemic agents through a prodigious variety of mechanisms. In the case of plants with high fiber content, the absorption of glucose can be delayed. For herbal remedies, however, this appears to be an unlikely mechanism of action. The volume consumed simply is not sufficient for such an effect. Other remedies can modify the gastrointestinal peptides implicated in the secretion of insulin. Some other possible mechanisms can imply alterations in insulin sensitivity or in the synthesis of insulin, the inhibition of insulin or its enzymatic interruption, interference with mitochondrial oxidation, or with gluconeogenesis [12,13].

With regard to phylogenetic distance (which extends from marine algae and fungi to the higher plants), among each of the families there is a strong indication of the great variety of active constituents, and chemotaxonomic studies are frequently utilized for the discovery of plants with novel active ingredients [14].

Prior to the discovery of treatments (synthetic) such as insulin and oral hypoglycemics, medicinal plants represented the predominant treatments for DM. The hypoglycemic remedies of plants continue to be frequent in developing countries where, to date, they are in use in the majority of cases and have been utilized for various centuries. These traditional treatments are also entertaining notable interest in Western nations. The evidence for these effects derive from studies with animals; notwithstanding this, the hypoglycemic effects in animal models are not necessarily transferable to humans, for clinical use, the data of assays on patients with diabetes and volunteers are essential [15].

Therefore, it is necessary to learn more on hypoglycemics that are natural in origin and on their mechanisms of action for the discovery of novel substances that can be carried out systematically. Among the species reported in the literature, only a small part of these have been experimentally and clinically evaluated to determine their efficacy [16]. On the other hand, it is noteworthy that some of the traditional remedies can be associated with a potential risk; for example, *Momordica charantia* has recently been described as being hepatotoxic in rats [15].

The great structural diversity of these substances explains the large variety that could be involved in the decrease in the variety of sugar in the blood. Some of these compounds may perhaps possess a considerable therapeutic potential, while others perhaps produce hypoglycemia as a collateral effect of its cytotoxicity, especially those that are hepatotoxic [16]; however, studies are needed to evaluate these natural compounds in different molecular targets involved in DM disease. Table 1 shows different groups of natural products with hypoglycemic effects isolated from Mexican plants, where flavonoids, aromatic compounds, and terpenoids are the most representative compound groups [10].

Among Mexican plants whose hypoglycemic effect has been demonstrated in different animal models, but whose active ingredients have not been isolated or characterized, we find the following: *Euphorbia prostate* [17], *Cuminum nigrum* [18], *Verbesina persicifolia*, *Psacalium decompositum* [19], and *Agrimonia eupatoria* [20].

### 1.2. Diabetes and Traditional Medicine in Mexico

In Mexico, although the use of Traditional Medicine is very frequent in different regions of the country, this medicine is mainly practiced in rural regions where the population is indigenous in its majority. The popular curative practices frequently constitute the sole health-care option; however, in addition, this practice is carried out in the great urban centers, such as Mexico City [62].

We know of the traditional indigenous medicine of Mexico through the customs of the currently marginated groups, where the use of the herbalism is fundamental, although mineral and animal products are also employed, all of the latter reinforced with religious devotions honoring their diverse deities [63].

In the XVI century, diseases such as diabetes were not known. The concerns of the traditional Mexican healers were the symptoms; thus, these were the sole aspects that the healers treated. Specifically with regard to diabetes, it remained unknown as metabolic insufficiency, and each of its manifestations continued to be treated separately. It was not until the end of the XIX and during the XX century that the pathology of the disease was described, beginning with the use of multiple therapies. Traditional Mexican Medicine focused its attention on the problem, but did not penetrate deeply into it. On the other hand, the use of herbalism in the home and in popular healing continued to cure only the patients’ symptomatology. It was throughout the XX century that academic medical knowledge on diabetes began to be incorporated into the popular medical culture and, thanks to the ethnobotanical investigation, the manner was registered in which the Mexican population referred and treated diabetes [63,64].

By the first third of the XX century, Dr. Maximino Martínez, in his book Plantas Medicinales de México, cited the following plants for diabetes: cuajilote; damiana; eucalyptus; matarique, and tronadora (trumpet flower). At mid-century, Dr. Luis G. Cabrera additionally recommends avocado, little by little, investigations on Mexican glycemic plants increased, and the Mexican National Institute of Indigenous Peoples (INI) cites many more, including cocoyol, prodigiosa, lengua de gallina (chicken tongue), lágrima de San Pedro, tejocote (Mexican Hawthorne fruit), gobernadora (greasewood), and tronadora [63,64].

In Mexico, persons suffering from diabetes have utilized preparations from traditional plants (Table 2 and Table 3), drinking a glass of the infusion of the juice (whether from the leaves or the stems, or from the fruit itself) before meals three times a day to obtain a hypoglycemic effect during the 5 or 6 h afterward [65]. Some of these plants utilized by the population are edible (Table 3); thus, two very important factors have merged in the treatment of the disease: their forming part of a good diet, and their possessing a hypoglycemic effect [8].

Among the edible plants that have shown experimentally to diminish blood glucose levels, we find cucumber (*Cucumis sativus*), chilacayote (*Cucurbita ficifolia*), cumin (*Cuminum cyminum*), nopal (prickly pear cactus, *Opuntia streptacantha*), bean (*Phaseolus vulgaris*), and spinach (*Spinacea oleracea*). A patient with diabetes should implement a dietary regimen utilizing edible plants and, in this manner, improve their diet and control, in part, the disease [87].

It is thought that a patient with this control potentially reduces the dose of hypoglycemic ingredients agent that the patient ingests; even patients with mild, non-insulin-dependent diabetes will avoid the use of these agents. Some of the reasons that support the use of edible plants is due to that synthetic hypoglycemic agents can have serious adverse effects, including hematological, cardiovascular, and gastrointestinal reactions, such as hypoglycemic and damage to the skin and liver; in addition, its use is not recommended during pregnancy. These non-edible plants utilized, such as antidiabetics, can entertain the same limitations, which does not occur with edible plants [87].

The importance of knowing the mechanism by which blood glucose levels diminish lies in that many compounds can be hepatotoxic agents that can exert an influence on the activity of some hepatic enzymes related with gluconeogenesis and that, at the moment of studying these, they can result in a false-positive result [16].

Aguilar and Xolalpa published, in the year 2002, a compilation of popularly utilized plants employed as hypoglycemic in Mexican population; these authors registered a total of 179 species belonging to 68 botanical families, among which the Asteraceae, Cactaceae, and Fabaceae families are the most representative. Similarly, these authors found that the leaves are the most used plant structures, followed by the stems and the roots. On this list, we find Matarique (*Psacalium decompositum*), that belongs to the Asteraceae family, which is also a subject of revision of this work.

### 1.3. Psacalium decompositum (A. Gray) H. Rob & Brettell

**Figure 1:**
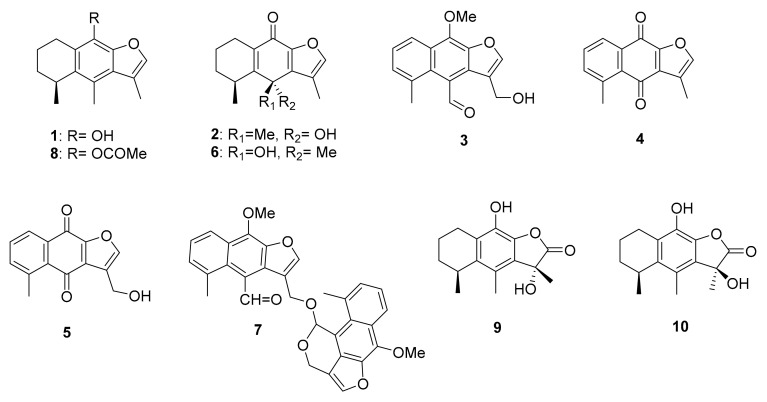
Sesquiterpenes isolated from *P. decompositum*: cacalol (**1**), cacalone (**2**), maturin (**3**), maturinone (**4**), maturone (**5**), epicacalone (**6**), dimaturin (**7**), cacalol acetate (**8**), 3-hydroxycacalolide (**9**), epi-3-hydroxycacalolide (**10**).

In Mexico, commonly used medicinal plants have been divided into complexes because they share characteristics in common, whether it be the name, the morphophysiological characteristics, the aromatic characteristics, or due to their traditional use. The Matarique complex comprises various species, among which we can highlight *Psacalium decompositum*, *Psacalium peltatum*, *Psacalium sinatum*, and *Acourtia thuberi*. *Psacalium decompositum* (*Cacalia decomposita*, *Odontotrichum decompositum*) is a perennial plant found in the pine-oak forests of the Sierra Madre Occidental mountain range in northeastern Mexico (Figure 1), where it is also known as Matarique or as pitcáwi (Tarahumara), a wild, land plant, in association with pine-oak forests. It grows from 30 cm–1.6 m in height. The stems are almost woody, densely hairy or hirsute at the base, with brown hairs. The simple basal leaves, alternating in rosettes, sub-orbicular, up to 40 cm in length, coriaceous in consistency, hirsute, with a deeply lobed margin, a peltate base, with a petiole (stalk), and with long, subpeltate cauline leaves that are smaller in size than the basal leaves. The flowers are hermaphroditic, with a sympetalous corolla, with the color of the flowers ranging from cream to brown. These flowers possess a thick fibrous rhizome. This plant is distributed in mountainous zones; however, it is best known in the Sierra Madre Occidental mountain range and in the center of the country of Mexico. This species originated in Mexico and grows in a semi-dry climate between 1950 and 2050 m above sea level (m asl) [97].

It is considered a threatened species. Surveys in the northern region of the country cite that its frequency has become noticeably reduced. Due to its hypoglycemic property, the species has been over-collected due to its commercialization and, at present, its populations are found in danger of extinction at the local level. With regard to the medical element, the aerial part of *Psacalium decompositum* is utilized to cure rheumatism, tumors, ulcers, fever, skin infections, toothache, diabetes, kidney diseases, and gastrointestinal and rheumatic pain. The root, in the form of a tea or an herbal infusion is employed against malaria, fever, diabetes, tumors, ulcers, rheumatism, kidney diseases, skin infections, and toothache. The crushed roots are utilized to treat snakebite, as well as for toothache, placing a piece of the root on the tooth with caries [97].

### 1.4. Bioactive Components of Psacalium decompositum

Román et al. (1992) demonstrated that the aqueous and methanolic extracts of the root present hypoglycemic activity. In the root, the following metabolites have been identified: cacalol (**1**), cacalone (**2**), maturin (**3**), maturinone (**4**), and maturone (**5**) (Figure 1), which are those most abundant in the sesquiterpenes in the root [48].

In 1998, Inman et al. described the properties of epicacalone (**6**), cacalone (**2**), cacalol (**1**) and dimaturin (**7**) (Figure 1) as hypoglycemic agents. The authors observed that these compounds administered by oral route significantly reduced plasma glucose levels employing a C57BL-6J ob/ob genetically obese and diabetic mouse model. These authors also developed a patent on the application of furanoeremophilanes and furanoeremophilane sesquiterpenes in the treatment of DM. Due to the fact that bioactive compounds usually are found in small amounts, it is convenient to carry out the chemical synthesis of these compounds; this is the case of cacalol (**1**) where more than ten methods of synthesis of this compound have been described. Thus, cacalol (**1**) can be obtained in the laboratory in gram quantities [98,99,100,101,102,103,104].

Alarcón-Aguilar et al., for their part in the year 2000, reported that the aqueous extract significantly reduces blood glucose levels dose-dependently in normal mice, administered intraperitoneally (i.p.). However, on testing the major sesquiterpene of the root cacalol (**1**), cacalone (**2**), and maturin (**3**), and the transformed product of cacalol (cacalol acetate) (**8**), the authors observed that these did not show a hypoglycemic effect on injecting them i.p. in the same model [48]. It is noteworthy that the contradictory results can be due to that each group employed a different animal model.

Prior studies conducted on *P. decompositum* have led to the isolation and structural studies of eremophilanes, which exert effects on the growth and development of *Amaranthus hypocondriacus* and *Echinocloa crusgalli* [105].

It has been observed that the aqueous acetate extracts were obtained from phytopathogenic fungi, principally in the growth of *Alternaria*, *Pythium*, *Fusarium*, and *Helminthosporium*. Thus, this has been proposed as an herbicide and as a natural selective fungicide [105].

The antimicrobial activity has also been proven of the metabolic, hexanic, and ethyl-acetate extracts of the root by means of the antibiogram method, finding that the extracts possess effects on *Candida albicans*, *Cryptococcus neoformans*, *Staphylococcus aureus*, and *Streptococcus pyogenes* [106]. Additionally, in the same study, it has demonstrated that among the hexanic extracts of the roots, cacalol (**1**) is the most abundant metabolite, and that also possess antimicrobial activity against *Staphylococcus aureus*, *Escherichia coli*, *Pseudomonas aeruginosa*, and *Proteus mirabilis* [106].

The chemical characterization of the metabolites contained in the roots and rhizomes of *P. decompositum* has demonstrated that their main composition consists of sesquiterpenoids such as cacalol (**1**), cacalone (**2**), maturin (**3**), epicacalone (**6**), 3-hydroxycacalolide (**9**), and epi-3-hydroxycacalolide (**10**) (Figure 1) [8,48]. Diverse studies report the hypoglycemic activity of the decoction of the roots [8], as well as of the fractions that contain fructooligosaccharides [78]. Notwithstanding this, as we have previously referred, DM is a complex disease in which the hypoglycemic action and the anti-inflammatory and antioxidant activities acquire great relevance, as do the possible hypolipidemic effects. Currently, studies have been initiated for the evaluation of these properties [107]. Reported a reduction in the levels of cholesterol and triglycerides, as well as a decrease in the body weight of rats utilized as experimental model when these were administered 150 mg/kg/day during 12 weeks of fructooligosaccharides obtained from the decoction of *P. decompositum* roots. In that same study, the authors observed novel anti-inflammatory properties due to the decrease in the levels of proinflammatory interleukins such as IL-6 and IL-1β, as well as the levels of IFN-γ, MCP-1, and VEGF, in which the latter are implicated in the development of insulin resistance and cardiovascular problems, among others. On the other hand, previous studies have evaluated the anti-inflammatory properties of the hexanic extract and its compounds cacalol (**1**) and cacalone (**2**), the latter obtained from the roots and rhizomes in two distinct assays: carrageenan-induced rat-paw edema, and TetradecanoPhorbol Acetate (TPA)-induced ear edema. Despite that in both assays the authors observed dose-dependent, anti-inflammatory effects, the hexane extract demonstrated significant action in the carrageenan test, while that of cacalone (**2**) was the most potent effect in both assays) [108]. These results coincide with those reported by Bakirel and coworkers [109], in which the methanolic extract of *P. decompositum* reduced the expression of the cytokines IL-1β, IL-6, and IL-8. In a recent report, the cacalol acetate, which is the most stable form of cacalol (**1**), was the object of an investigation with the aim of evaluating its anti-inflammatory properties and determining its possible participation in the NF-κB transcription factor signaling pathway. The results point out that the cacalol extract regulates the NF-κB signaling pathway, participating in the decrease of its activation, thus inhibiting a large number of inflammation mediators, thus reducing the inflammatory process in the generation of edema. In this manner, the participation of the natural products obtained from *P. decompositum* continues to contribute novel anti-inflammatory mechanisms that could establish the bases for new and future drugs [110].

Regarding the possible antioxidant properties of *P. decompositum*, of cacalol (1) as well as of cacalone (**2**), these have been reported as natural antioxidants, and a recent study indicates that cacalol (**1**) was capable of inhibiting the production of Reactive Oxygen Species (ROS) in bone marrow-derived mastocytes [111].

Nonetheless, in the case of *P. decompositum* and its sesquiterpenoids, and to our knowledge, the information on its antioxidant properties in diabetic animal models remains scarce.

### 1.5. Experimental Models for the Study of Plants with Hypoglycemic Activity

The most common models for inducing diabetes in experimentation animals are the treatments with Alloxan or Streptozotocin. Alloxan is a compound that possesses an affinity for ß cells, rendering it a good model for the study of DM. Notwithstanding this, it also presents some complications, such as the impossibility of establishing a relation between the dose of Alloxan and the effective concentration in the pancreas for the development of diabetes; it is difficult to determine the adequate concentration of Alloxan that inhibits the cells without producing necrosis [112].

Other commonly employed compounds for this purpose include the following: uric acid; dehydroascorbic acid; some quinolones; 2,4-dinitrophenol; diazoxide; some magnesium salts, and hormones such as epinephrine, glucagon, corticotropin, somatotropin, and pituitary extract [112].

The techniques used for the study of hypoglycemic activity in vivo are based in the use of normoglycemic and hyperglycemic animals (rat, mouse, and rabbit). Numerous techniques in vitro have been developed to determine the variety of mechanisms of action of the hypoglycemic agents discovered by means of assays in vivo. The following different aspects of the hypoglycemic response are commonly studied in vitro: the release of insulin from the pancreatic islets; the availability of peripheral insulin; the utilization of glucose, and the effect on hepatic enzymes [113].

Streptozocin causes the degeneration of pancreatic ß cells. This can be due to that Streptozocin induces an alteration in the mitochondrial function of the pancreatic islets. It is a compound of natural origin isolated from *Streptomyces achromogenes* [113].

The mechanism of some sulfonylureas at the cellular and subcellular levels has been determined through ß-cell cultures. Additionally, the role of the liver in diabetes has been studied in hepatocyte cultures of rat. Recently, human hepatomas have been used to study insulin receptors. Hikino and collaborators have determined a large number of mechanisms of hypoglycemic agents isolated from plants with a preparation of different hepatic enzymes [114].

### 1.6. Experimental Models for the Study of Plants with Anti-Inflammatory and Antioxidant Activity in Diabetes

Despite that hypoglycemic activity has been the focus-of-study for plants that have been traditionally utilized for the treatment of DM, at present there is ample evidence that shows the enormous complexity of the mechanisms involved in the pathogenesis and physiopathology of DM and its complications, such as the processes of chronic inflammation and oxidative stress [115]. Thus, in addition to the experimental models that evaluate the hypoglycemic activity of plants, in recent years models have been added that evaluate the anti-inflammatory and antioxidant properties of plants, with the purpose of providing integral information and of offering new knowledge on the possible and varied effects that the plants could supply on different targets-of-action for impacting a disease as complex as DM. In this manner, inflammation could be considered acute and chronic when the inflammatory response occurs in three distinct stages. The first stage is caused by an increase in the vascular permeability for the exudation of fluids in the interstitial space, while the second stage involves infiltrations of leukocytes from the blood into the tissues. The third stage refers to the formation of granuloma formation and tissue repair. The inflammatory response represents a complex biological and biochemical that involves immune-system cells and mediators. The mediators of the inflammatory processes are varied, but are generally recognized by ProstaGlandins (PG), LeukoTrienes (LTB4), Nitric Oxide (NO), the Platelet Activation Factor (PAF), bradykinins, serotonins, lipoxins, cytokines, and growth factors [116].

With respect to the antioxidant processes involved, recent evidence suggests that a sustained state of hyperglycemia can cause grave cellular damage through the oxidative stress, and that in a chronic progression, the latter could implicate the possible pathogenesis of the complications associated with DM. Thus, some authors suggest the identification of very specific biochemical changes caused by the hyperglycemia and that could be key, given the over-production of superoxide radicals. Therefore, these changes and their involved molecules represent possible targets-of-action for antioxidant molecules deriving from plants. These changes and possible targets-of-action comprise the following: an increase of fluid through the polyol pathway, reducing the levels of NADPH; an increase in the formation of the end products of Advanced Glycation End-products (AGE); an activation of protein kinase C, and an increase in the derivation of the excess of glucose through the Hexosamine Synthetic Pathway (HSP) [117].

Although there are a broad variety of reports in the literature on experimental animal models for the study of the anti-inflammatory and antioxidant properties of plants, it has only been during the past decade that the evaluations of these properties in experimental models of animals with induced diabetes were mainly initiated. The latter has the purpose of offering specificity and a panorama on DM, as well as overcoming the technical challenges of experimentation on working with plant-derived substances, such as the administration of extracts or factions, which are not always easy to manage in animal models. Thus, Table 4 attempts to contribute information on experimental models in diabetic animals utilized specifically for the evaluation of plant-derived natural products, such as extracts, fractions, or compounds. Therefore, subsequently, Table 4 presents the plant-derived substances that were evaluated, as well as the diabetic animal model employed.

## 2. Discussion

Due to DM is a disease that affects the worldwide population and exerts a great impact on diverse aspects, such as economic, social, familial, etc., information on matters of plants that yield new molecular structures with possible pharmacological effects for the treatment of DM is regarded as pressing. Among the plants that during the last decades have been studied in terms of their possible effects on DM, we find that *P. decompositum* is highlighted. The chemical characterization of the metabolites contained in the roots and rhizomes mainly consists of sesquiterpenoids such as cacalol (**1**), cacalone (**2**), maturin (**3**), epicacalone (**6**), 3-hydroxycacalolide (**9**), and epi-3-hydroxycacalolide (**10**) [8,48]. Cacalol as well as cacalone have exhibited potent hypoglycemic properties and the capacity of increasing insulin levels, as well as anti-inflammatory and antioxidant properties [78,108,111]. Thus, these sesquiterpenes are situated as the most outstanding of the species and result in being of utmost interest for continuing their study in possible models in vivo, in order to determine their potential integral pharmacological effects on DM. In this sense and given that the aqueous extract of *P. decompositum* has shown a hypoglycemic effect, the interaction of cacalol and cacalone with other components of the extracts and their possible potentialization, should also be explored. In this point, since the hypoglycemic effect of the *Matarique* involves also the presence of pyrrolizidine-type alkaloids, which are hepatotoxic components of different plant species [48], it is necessary to carry out toxicological evaluations in order to establish the mechanisms of action of these sesquiterpenes and guarantee their safety. Thus, modifications to the chemical structure of the sesquiterpenes isolated from *P. decompositum* could improve their properties on DM treatment, as well as reduce the original toxicity and adverse effects. 

On the other hand, cacalol is also an important antioxidant, very sensitive to oxidation due the presence of the alcohol group and furan ring [174]. Although an excess of antioxidants can produce a pro-oxidant effect, it has been proven that the acetylation of cacalol maintains the original scavenging of free radicals, which ensures its use as an antioxidant factor [175]. Additionally, cacalol acetate, more stable than cacalol, possesses anti-inflammatory properties that regulate the NF-ĸB signaling pathway through a decrease in the phosphorylation of IKB-α and p65, as well as the reduction in the expression of TNF-α, IL-1β, and IL-6, thus also offering an option in future interesting investigations, despite that a partial study did not exhibit hypoglycemic properties [48]. Nonetheless, as mentioned previously, perhaps its evaluation in more precise and updated models would offer new information. At any rate, the anti-inflammatory effect exhibited by cacalol acetate is *per se* an innovative mechanism of action [111] that requires more study, as does its possible combinations with other substances. On the other hand, given that *P. decompositum* forms part of a complex of plants denominated *Matarique* and that are traditionally utilized in Mexico for treating DM, it is interesting that another species belonging to such a complex, and of the same genus, *P. peltatum*, and from which an aqueous fraction was obtained with fructane content, has demonstrated an hypoglycemic effect and anti-inflammatory and antioxidant effects on Streptozotocin-induced diabetic mice [162] (Table 4). The latter situates it as a highly interesting species in the area of investigation in DM, due to this integral and candidate effect in later studies. In the same fashion, another species belonging to the same genus, *P. paucicapitatum*, a species endemic to Mexico, exhibited anti-inflammatory properties and a hypoglycemic effect in vivo on C57BL/6 mice when the aqueous extract with fructooligosaccharide content was evaluated [176].

Therefore, with the latter provided information, the species belonging to the *Psacalium* genus represents a potential hypoglycemic source with possible anti-inflammatory and antioxidant properties that could be utilizable in the future for the treatment of DM, and for which scientific information is scarce to date.

In addition, it is important to note that some triterpenes have been exerted a hypoglycemic effect, which prevent or help to prevent diabetic complications such as nephropathy or neuropathy, restoring the healing capacity [177]. This becomes highly relevant because uncontrolled blood glucose levels increase the chances of a viral infection, since DM is one of the main comorbidities [178] in the infection, development and worsening of COVID-19 [179]. Recent reports in China [180], Italy, and Mexico [181,182,183] have revealed that older patients with chronic diseases, especially DM, display a higher probability for contracting a severe condition in COVID-19 disease and that frequently, this leads to fatal consequences. Some of the mechanisms probably involved in the susceptibility to COVID-19 by patients with DM include: high affinity for binding the virus to the cell and efficient entry, decreased function of T cells, increased of susceptibility to hyperinflammation and cytokine storm syndrome, and the presence of cardiovascular diseases [184]. Consequently, the current pandemic caused by the SARS-CoV-2 virus, place the pharmacological agents available for treatment of DM as priority. In this way, with the absent of an effective treatment for COVID-19 and specially in DM patients, treatments using medicinal plants and their metabolites cannot be ruled out [185], either as enhancers of the immune system [186], as adjuvants in the management of diabetes [187] and as competitors/inhibitors of the receptors involved in SARS-CoV-2 infection [188]. Even more, recent reports indicated that terpenes could also inhibit some enzymes involved in the SARS-CoV-2 viral replication cycle [189].

In this sense, and one more time, as other authors have cited in previous studies, the study of Mexican plants for the treatment of DM continues to be found under development and is defining as many study approaches as strategies, which would imply more than one target, due not only to the complexity of the processes implicated in DM, as we have explained herein, but also because the possible future drugs with multiple actions could exert long-term impacts on other relevant aspects to consider, such as, for example, the increase in the adherence of the patient to the pharmacological treatment, but also an impact on the patient in terms of needing to purchase a sole drug, rather than many, and facilitation of control of comorbidities [10]. Therefore, once again, the study of Mexican plants for the treatment of DM stands out as an attractive source of compounds-to-explore, not only because of their registered ethnobotanical antecedents, but also because of the possibility of interaction on the distinct physiological targets implicated in DM in any of its stages, and the potential possibility of using these at the same time with other drugs that are currently prescribed for the treatment of DM. Table 4, which is elaborated in the present work, could be useful as a point of departure for the approach of future investigations that focus not only on the hypoglycemic properties of the plants, but also on the anti-inflammatory and antioxidant properties of the latter in relation to the treatment of DM.

## 3. Conclusions

The study of medicinal plants for the treatment of DM continues to be of great interest due to the possibility of the discovery of novel and diverse molecules that could be developed as utilizable therapeutic agents in this illness that exerts an impact worldwide. In the present review, the natural products obtained from plants are shown that have been employed in the treatment of DM, and particularly the plants used traditionally in Mexico, where DM is a serious health problem, as well as the anti-inflammatory and antioxidant experimental models in diabetic animals specifically utilized in plants that could be useful for future investigations of the multiple effects implicated in DM. Some species, such as *Psacalium decompositum*, whose extracts and isolated compounds, such as cacalol and cacalone, have revealed potent acute hypoglycemic effects and also influences on the increase of insulin, as well as anti-inflammatory and antioxidant effects, situate this species as a strong candidate for the development of a phytodrug useful in the treatment of DM. These sesquiterpenes are situated as the most outstanding of the species and result in being of utmost interest for continuing their study in possible models in vivo, in order to determine their potential integral pharmacological effects on DM. In the same way, to ensure the administration of cacalol and its derivatives, it is essential to deepen in toxicological studies considering the structural modifications for improvement the biological effects.

This information provides encouragement for future investigations that contribute to the understanding of these complex mechanisms and their interactions, as well as its also being potentially able to establish the bases for the development of a new type of drugs that are effective in DM.

## Figures and Tables

**Table 1:** Natural hypoglycemic products isolated from Mexican plants.

Chemical Type	# Active Molecules	Subtype	Reference
Flavonoids	28	Flavonols	[21,22,23,24,25,26]
		Flavones	[27,28,29,30,31]
		Dihydrochalcones	[32,33]
		Biflavone	[30]
		Flavanone	[34]
		Flavan-3-ols	[30,35,36,37]
Aromatic compounds	25	Coumarins	[38,39]
		Hydroxycinnamic acids	[28,35,40,41]
		Chromones	[24,42]
		Depsides	[43]
		Phthalides	[44]
		α-pyrone glycosides	[38,39]
		Stilbene	[45]
		Hydroxybenzoic acid	[35]
Terpenoids	23	Sesquiterpenes	[8,28,42,46,47,48,49,50,51]
		Diterpene	[30]
		Limonoids	[52,53]
		Cucurbitane	[54]
		Oleananes	[55,56,57]
		Ursarnes	[58,59]
Steroids	4		[29,54]
Oligosaccharides	4		[60]
Polyalcohol	1		[61]

# (number) of active molecules is considered according to the report of the studies of 40 hypoglycemic Mexican plants.

**Table 2:** Mexican plants with experimentally demonstrated hypoglycemic activity.

Scientific Name	Botanical Family	Common Name	Used Part of the Plant for Infusion	Reference
*Acourtia thurberi*	Asteraceae	Matarique	Root	[8,46,66]
*Bauhinia divaricata*	Fabaceae	Pezuña de vaca	Leaves	[67]
*Bidens odorata*	Asteraceae	Aceitilla	Whole Plant	[68]
*Buddleia americana*	Scrophulariaceae	Tepozán	Leaves	[67]
*Calea zacatechichi*	Asteraceae	Prodigiosa	Stem, Leaves and Root	[67]
*Cecropia obtusifolia*	Cecropiaceae	Guarumbo	Leaves	[9,40,65,69,70]
*Coix lacryma*	Poaceae	Lágrimas de San Pedro	Stem and Leaves	[67]
*Crataegus pubescens*	Rosaceae	Tejocote	Root	[67,71]
*Cynodon dactylon*	Poaceae	Grama	Stem and Leaves	[67,72]
*Eriobotrya japonica*	Rosaceae	Níspero	Leaves	[65]
*Euphorbia prostrata*	Euphorbiaceae	Golondrina	Whole Plant	[17,64]
*Guaiacum coulteri*	Zygophyllaceae	Guayacan	Stem	[67]
*Guazuma ulmifolia*	Malvaceae	Guacima	Leaves	[64,73,74]
*Lepechinia caulescens*	Lamiaceae	Salvia	Flowers	[64,65,75]
*Marrubium vulgare*	Lamiaceae	Marrubio	Stem, Leaves and Root	[67,76]
*Musa sapientum*	Musaceae	Plátano	Fresh Flowers	[64]
*Psacalium decompositum*	Asteraceae	Matarique	Root	[48,77,78,79]
*Psacalium peltatum*	Asteraceae	Matarique	Root	[65,80,81,82]
*Psittacanthus calyculatus*	Loranthaceae	Muérdago	Stem, Leaves and Flowers	[83]
*Rhizophora mangle*	Rhizophoraceae	Mangle rojo	Stem	[37,64]
*Salpianthus macrodonthus*	Nyctaginaceae	Catarinilla	Stem and Leaves	[65]
*Solanum verbascifolium*	Solanaceae	Malabar	Stem and Leaves	[65]
*Tecoma stans*	Bignoniaceae	Tronadora	Stem and Leaves	[65,74,84]
*Teucrium cubense*	Lamiaceae	Agrimonia	Stem and Leaves	[65,84]
*Tournefortia hirsutissina*	Heliotropiaceae	Lágrimas de San Pedro	Stem	[64,85]
*Trigonella foenum-graecum*	Fabaceae	Paracata	Leaves	[64,86]
*Turnera diffusa*	Passifloraceae	Damiana	Leaves	[49,64]

**Table 3:** Edible Mexican plants with experimentally demonstrated hypoglycemic activity.

Scientific Name	Botanical Family	Common Name	Used Part of the Plant	Reference
*Cuminum cyminum*	Apiaceae	Comino	Seed Infusion	[87,88]
*Cucumis sativus*	Cucurbitaceae	Pepino	Fruit Juice	[87,89]
*Cucurbita ficifolia*	Cucurbitaceae	Chilacayote	Fruit Juice	[87,90,91,92,93]
*Opuntia streptacantha*	Cactaceae	Nopal	Stem Juice	[87,94,95]
*Phaseolus vulgaris*	Fabaceae	Fríjol	Sheath Infusion	[87,96]
*Spinacea oleracea*	Amaranthaceae	Espinaca	Leaves Juice	[87]

**Table 4:** Experimental models for the study of the anti-inflammatory and antioxidant properties of plants related with DM.

Biological Target	Name of the Assay	Plant Species/Part of the Plant Used or Active Molecule	Diabetic Animal Model/Doses of the Plant Used	Reference
Acute inflammation	Carrageenan and histamine-induced paw edema	*Passiflora edulis* (Passifloraceae)/Flour fruit peel;	Alloxan induced diabetic mice/0.5–25 mg/kg;	[118,119,120,121]
		*Harungana madagascariensis*	Alloxan induced diabetic rats/25, 50 and 100 mg/kg;	
		(Hypericaceae)/Stem-bark ethanolic extract;	STZ induced diabetic rats/100 mg/kg;	
		*Eugenia uniflora*(Myrtaceae)/Methanolic extract of leaves;	STZ induced diabetic mice/25–1600 mg/kg;	
		*Sclerocarya birrea* (Anacardiaceae)/Aqueous stem-bark;		
	Xylene-induced ear edema thickness and weight	*Typha orientalis* (Typhaceae)/Polysaccharides of pollen;	STZ induced diabetic rats/0.1, 0.2 and 0.4 g/kg;	[122,123]
		*Pyrus bretschnrideri, P. communis, P. ussuriensis (Rosaceae)/*Peel and pulp	STZ induced diabetic mice/500 mg/kg	
	Myeloperoxidase (MPO)	Kaempferol-3,7-O-(α)-dirhamnoside;	Alloxan induced diabetic rats/50, 100 and 200 mg/kg;	[124,125,126]
		*Withania coagulans* (Solanaceae)/Aqueous fruit extract;	STZ induced diabetic rats/10 mg/kg;	
		Oleuropein/	Alloxan induced diabetic rats/15 mg/kg	
Chronic inflammation	Cotton pellet-induced granuloma	*Zingiber officinale (Zingiberaceae)/*Aqueous extract; *Bridelia micrantha* (Phyllantaceae)/Methanolic extract of leaves	STZ induced diabetic mice/100, 200 and 400 mg/	[127,128]
			100 mL;	
			STZ induced diabetic rats/100, 200 and 400 mg/kg	
Antioxidant in vitro activity	Diphenyl-picryl-hydrazyl radical scavenging	*Pyrus bretschnrideri, P. communis, P. ussuriensis (Rosaceae)/*Peel and pulp;	STZ induced diabetic mice/500 mg/kg;	[123,124,129,130,131]
	(DPPH)	Kaempferol-3,7-O-(α)-dirhamnoside;	Alloxan induced diabetic rats/50, 100 and 200 mg/kg; Alloxan induced diabetic rats/200 and 500 mg/kg; Alloxan induced diabetic rats/80 mg/kg;	
		*Cyperus rotundus* (Cyperaceae)/Ethanol extract of rhizomes; *Cecropia pachystachya* (Urticaceae)/Methanol extract of leaves;	STZ induced diabetic mice/200 mg/kg	
		*Smallanthus sonchifolius* (Asteraceae)/Tuber extract and chlorogenic acid		
	Oxygen radical absorbance capacity (ORAC)	*Eugenia uniflora* (Myrtaceae)/Aqueous extract;	NOD mice/0.06 g/100 mL;	[132,133,134]
		*Passiflora alata* (Passifloraceae)/Aqueous leaves extract;	NOD mice/15 g leaf/L;	
		Grape pomace extract	STZ induced diabetic rats/400 mg/kg	
	Trolox equivalent antioxidant capacity (TEAC)	*Lycium barbarum* (Solanaceae)/Fruit water decoction;	Alloxan induced diabetic rabbits/0.25 g/kg and 10 mg/kg; db/db (+/+) C57BL/KsL mice/5% of the diet	[135,136]
		garlic and aged black garlic/water extract		
	Ferric reducing antioxidant power (FRAP)	*Morus alba* (Moraceae)/leaves;	STZ induced diabetic rats/6 and 22 mg/g HF diet; NOD mice/15 g leaf/L;	[132,133,137]
		*Passiflora alata* (Passifloraceae)/Aqueous leaves extract;	NOD mice/0.06 g/100 mL	
		*Eugenia uniflora* (Myrtaceae)/Aqueous extract;		
	Superoxide anion radical scavenging (SOD)	*Annona squamosal (*Annonaceae)/Aqueous extract*;*	STZ induced diabetic rats/300 mg/kg;	[138,139,140]
		*Calotropis gigantea* (Asclepiadaceae)/chloroform extracts of leaf and flower; *Emblica officinalis*	STZ induced diabetic rats/10, 20 and 50 mg/kg;	
		(Phyllanthaceae)/Hydromethanolic extract of leaves	STZ induced diabetic rats/100, 200, 300 and 400 mg/kg	
	Hydroxyl radical scavenging	*Cyperus rotundus* (Cyperaceae)/Hydroethanolic extract;	Alloxan induced diabetic rats/200 and 500 mg/kg;	[129,141]
		*Moringa oleifera* (Moringaceae)/Ethanolic leaf extract	C57BLKS/J Iar-+Leprdb/+Ledprdb mice/150 mg/kg	
	Nitric oxide radical scavenging	*Euphorbia hirta* (Euphorbiaceae)/Ethanolic and petroleum ether flower extracts;	Alloxan induced diabetic mice/250 and 500 mg/kg;	[142,143]
		*Syzygium mundagam* (Myrtaceae)/Petroleum ether, ethyl acetate, methanol and hot water extracts of bark	STZ induced diabetic rats/250 and 500 mg/kg	
	Total phenolic content	Grape pomace extract;	STZ induced diabetic rats/400 mg/kg;	[134,144]
		*Euphorbia hirta* (Euphorbiaceae)/Petroleum ether, chloroform and ethyl acetate extracts of aerial parts	STZ induced diabetic mice/500 mg/kg	
	Metal chelating activity	*Syzygium mundagam* (Myrtaceae)/Petroleum ether, ethyl acetate, methanol and hot water extracts of bark;	STZ induced diabetic rats/250 and 500 mg/kg;	[143,145]
		*Cinnamomum tamala* (Lauraceae)/Oil of leaves	STZ induced diabetic rats/100 and 200 mg/kg	
	Hydrogen peroxide (H_2_O_2_) radical scavenging	*Achyranthes aspera* (Amaranthaceae)/Ethanolic extract of stem and leaves;	Alloxan induced diabetic mice/200 and 400 mg/kg;	[145,146]
		*Cinnamomum tamala* (Lauraceae)/Oil of leaves	STZ induced diabetic rats/100 and 200 mg/kg	
	Reducing power (RP)	*Euphorbia hirta* (Euphorbiaceae)/Ethanolic and petroleum ether flower extracts;	Alloxan induced diabetic mice/250 and 500 mg/kg; Alloxan induced diabetic mice/200 and 400 mg/kg	[142,146]
		*Achyranthes aspera* (Amaranthaceae)/Ethanolic extract of stem and leaves		
	Total flavonoid	Grape pomace extract;	STZ induced diabetic rats/400 mg/kg;	[134,147,148]
		*Hybanthus enneaspermus* (Violaceae)/Alcoholic extract;	STZ- induced diabetic rats/250 and 500 mg/kg;	
		*Aloe barbadensis* (Asphodelaceae)/Ethanolic skin leaves extract	STZ- induced diabetic rats/1.25 g/kg	
	Xanthine oxidase	*Croton cajucara* (Euphorbiaceae)/Aqueous extract of bark;	STZ- induced diabetic rats/1.5 mL i.g.;	[149,150]
		*Pimpinella tirupatiensis* (Apiaceae)/Aqueous extract of tuberous root		
			STZ- induced diabetic rats/750 mg/kg	
	Conjugated diene	*Helicteres isora* (Sterculiaceae)/Aqueous extratc of bark;	STZ- induced diabetic rats/100 and 200 mg/kg;	[151,152]
		*Salmalia malabarica* (Malvaceae)/Hydromethanolic extract of sepals	STZ- induced diabetic rats/20 mg/0.5 mL distilled water/100 g	
	Phosphomolybdenum method	*Scoparia dulcis* (Plantaginaceae)/Ethanolic extract of aerial parts;	Alloxan induced diabetic mice/100 and 200 mg/kg; Alloxan induced diabetic rats/ppm	[153,154]
		*Satureja khuzestanica (Lamiaceae)/Oil of aerial parts*		
	Cytochrome tests	*Averrhoa bilimbi (Oxalidaceae)/Aqueous soluble, butanol soluble, ethyl acetate and hexane fractions of ethanolic leaf extract;*	STZ- induced diabetic rats/125 mg/kg;	[155,156]
		*Averrhoa bilimbi (Oxalidaceae)/Aqueous and butanol f*ractions of ethanolic leaf extract		
			STZ- induced diabetic rats/125 mg/kg;	
	Erythrocyte ghost systems	α-eleostearic acid and punicic acid;	STZ- induced diabetic rats/0.5% of the total lipid given for each isomer;	[157,158]
		*Sesbania grandiflora* (Fabaceae)/Methanolic extract of flowers	STZ- induced diabetic rats/250 mg/kg	
	Ferric thiocyanate (FTC)	*Momordica charantia* (Cucurbitaceae);	STZ- induced diabetic rats/20 mg/kg;	[159,160]
		Aqueous extract of the fruit; *Cleome rutidosperma* (Cleomaceae) and *Senecio biafrae* (Asteraceae)/Petroleum ether, acetone, ethanol, and aqueous extract of aerial parts	STZ- induced diabetic mice/500 mg/kg	
	Thiobarbituric acid (TBARs)	*Morus alba* (Moraceae)/leaves; *Morus alba;* (Moraceae)/Hydroethanolic extract of leaves;	STZ- induced diabetic rats/0.25, 0.5 and 1 g/kg;	[161,162,163,164]
		*Linum usitatissimum* (Linaceae)/Aqueous extract of the seed;		
		*Alnus nitida (Betulaceae)/*Methanol, hexane, chloroform, ethyl acetate and soluble residual aqueous fractions of leaves	STZ induced diabetic rats/6 and 22 mg/g HF diet; Alloxan induced diabetic mice/1 mL of extract;	
			Alloxan induced diabetic rats/100 and 200 mg/kg	
Antioxidant in vivo activity	Reduced GSH activity	*Emblica officinalis*	STZ induced diabetic rats/100, 200, 300 and 400 mg/kg;	[125,140]
		(Phyllanthaceae)/Hydromethanolic extract of leaves;	STZ induced diabetic rats/10 mg/kg	
		*Withania coagulans* (Solanaceae)/Aqueous fruit extract		
	Estimation of MDA	*Teucrium polium* (Lamiaceae)/Aqueous extract;	STZ- induced diabetic rats/0.5 g/kg;	[125,161,162]
		*Withania coagulans* (Solanaceae)/Aqueous fruit extract;	STZ induced diabetic rats/10 mg/kg;	
		*Psacalium peltatum* (Asteraceae)/Aqueous fraction with fructan content of roots extract	STZ induced diabetic mice/200 mg/kg	
	Ferric reducing ability of plasma	*Nigella sativa* (Ranunculaceae)/Methanolic extract of seed, and oil;	Alloxan induced diabetic rats/270 and 810 mg/kg; 2.5 mL/kg of oil;	[163,164]
		*Camellia sinensis (Theaceae)/*Hydromethanolic extract	STZ- induced diabetic rats/3 mg/L	
	Catalase (CAT)	*Calotropis gigantea* (Asclepiadaceae)/Chloroform extracts of leaf and flower;	STZ induced diabetic rats/10, 20 and 50 mg/kg;	[139,165,166,167]
		*Bacopa monnieri* (Plantaginaceae)/Hydroethanolic extract of aerial parts;		
		*Toddalia asiatica* (Rutaceae)/Hexane, ethyl acetate and methanol extract of leaves;	STZ induced diabetic rats/125 and 250 mg/kg;	
		*Coffea arabica* (Rubiaceae)/aqueous extract of green beans		
			STZ induced diabetic rats/250 and 500 mg/kg;	
			STZ induced diabetic01 rats/50 mg/kg	
	Glutathione reductase (GR)	*Punica granatum (*Lythraceae)/*Peels extract;*	STZ- induced diabetic rats/10 and 20 mg kg^−1;^	[168,169]
		*Zingiber officinale* (Zingiberaceae)/Ethanolic extract		
			STZ- induced diabetic rats/200 mg/kg	
	Lipid peroxidation (LPO)	*Rosmarinus officinalis* (Lamiaceae)/Ethanolic extract;	STZ- induced diabetic rabbits/50, 100 and 200 mg/kg;	[170,171]
		*Phyllanthus niruri* (Phyllanthaceae)/Aqueous extract of leaves	STZ- induced diabetic rats/200 and 400 mg/kg	
	LDL assay	*Bauhinia orficata* (Fabaceae)/Aqueous, hexane and methanol extract of leaves;	Alloxan induced diabetic rats/200 and 400 mg/kg;	[172,173]
		*Vernonia amigdalina* (Asteraceae)/Aqueous extract of leaves	STZ- induced diabetic rats/200 mg/kg	

## Data Availability

Not applicable.
